# External validation of the parental attitude about childhood vaccination scale

**DOI:** 10.3389/fpubh.2023.1146792

**Published:** 2023-05-15

**Authors:** Ramy Mohamed Ghazy, Sally Waheed Elkhadry, Suzan Abdel-Rahman, Sarah Hamed N. Taha, Naglaa Youssef, Abdelhamid Elshabrawy, Sarah Assem Ibrahim, Salah Al Awaidy, Tareq Al-Ahdal, Bijaya Kumar Padhi, Noha Fadl

**Affiliations:** ^1^Tropical Health Department, High Institute of Public Health, Alexandria University, Alexandria, Egypt; ^2^Epidemiology and Preventive Medicine Department, National Liver Institute, Menoufia University, Menoufia, Egypt; ^3^Biostatistics and Demography Department, Faculty of Graduate Studies for Statistical Research, Cairo University, Giza, Egypt; ^4^Forensic Medicine and Clinical Toxicology Department, Faculty of Medicine, Cairo University, Giza, Egypt; ^5^Department of Medical-Surgical Nursing, College of Nursing, Princess Nourah Bint Abdulrahman University, Riyadh, Saudi Arabia; ^6^Health Affairs, Ministry of Health, Muscat, Oman; ^7^Institute of Global Health (HIGH), Heidelberg University, Heidelberg, Germany; ^8^Community Medicine and School of Public Health Department, Postgraduate Institute of Medical Education and Research, Chandigarh, India; ^9^Family Health Department, High Institute of Public Health, Alexandria University, Alexandria, Egypt

**Keywords:** external validation, calibration, discrimination, parental attitude about childhood vaccination, seasonal influenza vaccine

## Abstract

**Introduction:**

Internal validation techniques alone do not guarantee the value of a model. This study aims to investigate the external validity of the Parental Attitude toward Childhood Vaccination (PACV) scale for assessing parents’ attitude toward seasonal influenza vaccination.

**Methods:**

Using a snowball sampling approach, an anonymous online questionnaire was distributed in two languages (English and Arabic) across seven countries. To assess the internal validity of the model, the machine learning technique of “resampling methods” was used to repeatedly select various samples collected from Egypt and refit the model for each sample. The binary logistic regression model was used to identify the main determinants of parental intention to vaccinate their children against seasonal influenza. We adopted the original model developed and used its predictors to determine parents’ intention to vaccinate their children in Libya, Lebanon, Syria, Iraq, Palestine, and Sudan. The area under the curve (AUC) indicated the model’s ability to distinguish events from non-events. We visually compared the observed and predicted probabilities of parents’ intention to vaccinate their children using a calibration plot.

**Results:**

A total of 430 parents were recruited from Egypt to internally validate the model, and responses from 2095 parents in the other six countries were used to externally validate the model. Multivariate regression analysis showed that the PACV score, child age (adolescence), and Coronavirus disease 2019 (COVID-19) vaccination in children were significantly associated with the intention to receive the vaccination. The AUC of the developed model was 0.845. Most of the predicted points were close to the diagonal line, demonstrating better calibration (the prediction error was 16.82%). The sensitivity and specificity of the externally validated model were 89.64 and 37.89%, respectively (AUC = 0.769).

**Conclusion:**

The PACV showed similar calibration and discrimination across the six countries. It is transportable and can be used to assess attitudes towards influenza vaccination among parents in different countries using either the Arabic or English version of the scale.

## 1. Introduction

Influenza viruses are a major threat to global health, causing high rates of morbidity and mortality (1). Influenza burden fluctuates from year to year, depending on which viruses are circulating and the number of people infected. The severity of illness can range from mild to severe. High-risk groups, such as pregnant women, young children, older adults, and those with chronic medical conditions or compromised immune systems, are more likely to develop complications (2). Globally, seasonal influenza infects up to 20% of the population during winter, resulting in up to 650,000 annual deaths from influenza-related respiratory diseases. Furthermore, influenza has a large economic impact in terms of expenses and lost working hours (3).

Influenza viruses infect the nose, throat, and lungs and are easily transmitted among children because they frequently touch their nose, eyes, and mouth and touch each other while playing (3, 4). Furthermore, there are many interactions between parents/caregivers and children, including holding hands, picking up, feeding, changing diapers, and other activities (4). Influenza continues to have a serious impact on the morbidity and mortality of children and continues to increase annually. Young children, children with chronic illnesses, and household members are more vulnerable to influenza-related illnesses. Despite this, seasonal influenza vaccination rates in childhood remain low (5, 6).

The most vital step in preventing seasonal influenza infection is vaccination (3, 7). A crucial first step in avoiding the spread of influenza among healthcare workers (HCWs), patients, high-risk populations, and children is to increase vaccination rates. Recently, the Centers for Disease Control and Prevention (CDC) recommended that all people aged 6 months and older, including HCWs, patients, and residents of long-term care homes, should receive vaccinations unless otherwise specified (7). Seasonal influenza vaccination is available in the fall and provides protection during the influenza season (November to April) (4). Full protection from the moment of vaccine administration typically takes 2 weeks. Children under the age of 9 years require two doses 4 weeks apart during the first year of immunization. Seasonal influenza vaccines are not recommended for infants below 6 months as breast milk protects against various respiratory infections, including influenza (8). In addition, their safety and immunogenicity have not yet been approved, especially if administered with other vaccines (9).

Despite the fact that yearly influenza vaccination is recommended for all children aged 6 months to 18 years, little is known about the level of parental hesitation to vaccinate their children against seasonal influenza worldwide (10). The World Health Organization (WHO) lists vaccine hesitancy (VH) as one of the leading 10 causes of global health threats in 2019 (11). VH refers to a delay in acceptance or refusal of immunization, despite the availability of vaccination services. VH is complicated and varies across time, region, and type of vaccine. It is affected by complacency, constrain, and confidence in the vaccine and its service delivery (12).

The Parent Attitudes about Childhood Vaccines (PACV) survey is a reliable technique that has been successfully used in several countries to identify parental VH (13, 14). The PACV has been internally validated in English and Arabic versions (10, 13, 15). Unlike internal validation, external validation aims to assess the performance of a risk-prediction model for new individuals (new dataset). External validation aims to investigate whether the developed model can accurately predict similar but distinct individuals outside the original setting (16). It reveals the degree of heterogeneity and the extent to which the model can be generalized outside the development set. External validation measures the predictive accuracy of the developed model under different circumstances, indicating its transportability to other individuals at different times (temporal validation) or in different countries (geographic validation) (16, 17). A model is said to be “transportable” if it continues to perform well in a population distinct from the one for which it was initially designed (18).

Few studies have evaluated external validation (17, 19, 20). However, it is insufficient to validate the prediction model internally and indicate its success in predicting the outcome of interest. Furthermore, internal validation techniques alone do not guarantee the value of a model (21, 22). Assessing the external validity of prediction models is vital to verify their performance in other samples, as they usually perform better in development samples than in other new or different samples. Thus, in addition to assessing internal validity, the performance of prediction models should be validated in new individuals before use in practical studies. We hypothesized that the PACV is valid for the assessment of parental attitude toward vaccination. In this context, this study aimed to externally validate the PACV scale in six countries in the Eastern Mediterranean Region (EMR).

## 2. Materials and methods

### 2.1. Study design

A cross-sectional survey was conducted from September 8 to October 15, 2022, among the general population from seven countries in EMR using an online questionnaire.

### 2.2. Study population and sampling methods

The snowball sampling approach was used to include individuals who met the following eligibility criteria: parents aged 18 or older, with one or more children aged 6 months to 19 years, with a mobile phone or computer, who were able to self-complete the survey, and who resided in one of the following randomly selected EMR countries (Egypt, Libya, Lebanon, Syria, Iraq, Palestine, Sudan).

### 2.3. Data collection

The survey was created using Google Forms and distributed in two language versions (English and Arabic) via social media channels (Facebook and Twitter), WhatsApp, and emails. Before data collection, a pilot test was conducted to assess the feasibility and comprehensibility of the questionnaire. The feasibility and accessibility of the web application were also evaluated. Each data collector was asked to provide at least two responses to calculate the time required to complete the survey. The time spent by the respondents to fill in the questionnaire was 5–12 min. Only minor edits, including linguistic corrections, were made based on the respondents’ recommendations (Supplementary File 1).

### 2.4. External validation process

#### 2.4.1. Development of the PACV questionnaire in the original sample

The development and scoring technique of the PACV have been explained elsewhere (13). In summary, 230 parents of children aged 19–35 months were recruited for this study. The domains of PACV (safety, efficacy, and attitude and behavior) explained 70% of parental intention to vaccinate children. The Cronbach’s alphas for the three domains were 0.74, 0.84, and 0.74, respectively. The overall PACV score was calculated by adding the weighted scores for each item. The total score ranges from 0 to 100 points. Participants were classified as hesitant (≥50 points) or non-hesitant (<50 points). The equation for the developed model was not available. Therefore, the internal validity of the PACV was tested to develop an equation that could be used for external validation.

#### 2.4.2. Steps of external validation of PACV

We collected data from seven countries to verify the geographic validation of the PACV tool for predicting parents’ intention to vaccinate their children against seasonal influenza. First, we estimated the predictors of parents’ intention to vaccinate their children using data collected from Egypt (internal validation). Second, we adopted the original model developed in the first step and utilized its predictors to predict parents’ intention to vaccinate their children in the remaining countries: Libya, Lebanon, Syria, Iraq, Palestine, and Sudan (external validation). The predictive performance of the adopted model was measured when it was applied to the second group by quantifying the main aspects of discrimination and calibration. We followed the Transparent Reporting of a Multivariable Prediction Model for Individual Prognosis or Diagnosis (TRIPOD) Statement, a reporting guideline for research creating or validating a multivariable prediction model (Supplementary File 2).

### 2.5. Sample size calculation

Internal validation requires a minimum sample size of 300 as a rule of thumb (23), and our study exceeded this requirement with a sample size of 430 in the internal validity group. The predicted PACV model was developed using data from Egypt.

For external validation, it is recommended to have at least 100 events and 100 non-events to ensure accurate and precise estimates of performance measures, and even larger sample sizes (a minimum of 200 events and 200 non-events) to derive flexible calibration curves (24, 25). To validate the PACV tool externally in six countries, we collected data from 2095 respondents, with 399 cases in Libya, 301 cases in Lebanon, 386 cases in the Syrian Arab Republic, 389 cases in Iraq, 389 cases in Palestine, and 231 cases in Sudan. We chose to develop the predicted model using data from Egypt because we had previously internally validated the Arabic version of the PACV tool using Egypt’s data in a previous publication, which indicated the validity and reliability of the PACV instrument in Arabic language (15).

### 2.6. Statistical analysis

Logistic regression was used to predict parents’ intention to vaccinate their children, using multiple predictor variables, including the PACV score. The outcome variable is binary, “one” for parents who intended to vaccinate their children against seasonal influenza and “zero” for parents who did not intend to vaccinate their children against seasonal influenza. Binary logistic regression was used to calculate the predicted outcomes. The probability equation for the logistic prediction model takes the following form. $p\left(probability\right)=\frac{1}{1+e^{-\left(\beta_{0}+PI\right)}}$

where $\beta_{0}$ = the intercept and the prognostic index (PI) = $\sum_{i=1}^{n}\beta_{i}X_{i},$ $\beta =\ln\;\left(oddsratio\right)$ .

The prognostic index (PI) is the main component of the prediction equation. The PI is a linear predictor calculated by summing the model’s predictors ( $X_{s}$ ) multiplied by their regression coefficients $(\beta_{i}).X$ s denotes the independent variables that include the PACV score and the characteristics of both parents and children. To develop an accurate predictive model, we checked logistic regression assumptions before running the test. We investigated the linearity between the PACV score, mother’s age, number of children, birth order, and logit of parents’ intention outcomes. Smoothed scatter plots indicated that all continuous variables were relatively linearly associated with parents’ intention on the logit scale (Supplementary Figure S1). Cook’s distance and standardized residuals were measured to check the influential values. Cook’s distance was used to determine the most extreme observations (Supplementary Figure S2). However, we did not have influential observations because their absolute standardized residuals were lesser than three, as indicated in Supplementary Figure S3. Moreover, our model was free from multicollinearity because the variance inflation factor was <5.

To assess the internal validity of the model, we utilized a more advanced machine learning technique and resampling methods to select various samples and refit the model for each sample. Fitting the developed model to each new sample produced additional information regarding the variability of the developed model’s fit. We used the most common methods known as cross-validation techniques. The samples were selected using leave-one-out cross-validation (LOOCV) and K-Fold Cross-Validation (K-fold CV) techniques. The average prediction error was then estimated from the drawn samples to assess the performance of the developed model (26). Further details can be found in Supplementary File 3.

To evaluate the external validity of the predictive model, we used common assessment metrics and methods. First, we computed the prediction accuracy rate, which refers to the proportion of correctly predicted observations. Conversely, the prediction error rate refers to the proportion of incorrectly predicted observations. Confusion matrices were used to determine the proportions of type I and type II errors. Type I errors occur when the predictive model incorrectly predicts parents who do not intend to vaccinate their children in the intending group. In contrast, type II error refers to incorrectly assigning parents in the intending group to the group that includes parents who lack the intention of vaccination.

Furthermore, we measured the sensitivity and specificity metrics that summarize the model’s overall performance. The sensitivity of the predicted model was measured using the true-positive rate (TPR), which is the proportion of parents in the intended group correctly predicted by the developed model. For comparison, the specificity of the predicted model was measured using the true-negative rate (TNR), which is the proportion of parents in the non-intending group who were correctly predicted by the developed model. Therefore, the false-positive rate (FPR) is the proportion of parents in the non-intending group that are incorrectly predicted in the intending group. The FPR is the complement of specificity.

To visualize the predictive model performance, we used the receiver operating characteristic area under the curve (ROC-AUC), which shows the sensitivity against “1-specificity” at various values of the probability cutoff. The AUC was calculated to summarize the overall performance of the predictive model, which indicates the ability of the model to distinguish events from non-events (i.e., discrimination). Additionally, we visually compared the observed and predicted probabilities of parents’ intention to vaccinate their children using a calibration plot. The 45° line indicates perfect agreement between the predicted and observed probabilities (calibration). Overprediction results in points above the diagonal line, whereas underprediction results in points below the diagonal line.

### 2.7. Considerations of ethics

This study was part of a larger project that aimed to evaluate parents’ seasonal influenza vaccine hesitancy in the EMR (27). The Ethics Committee of the Faculty of Medicine at Alexandria University, Egypt approved this study (IRB no. 0305688). The study followed the Helsinki Declaration and the Ethics Committee guidelines to ensure anonymity, confidentiality, and voluntary participation. All participants provided written informed consent before taking part in the study. The collected information was stored in a coded format on a secure computer that was only accessible to the principal investigator.

## 3. Results

### 3.1. Groups used to develop and validate the model

Table 1 shows the differences in average values of other predictors, including parents’ socio-demographic characteristics (mother age, education, employment, residence, and parents’ previous influenza vaccination) and child characteristics (child’s birth order, whether the child had a chronic disease, whether the child got sick from influenza last year, whether the child got influenza vaccination last year, and whether the child got routine vaccination), between the development and validation samples. Notably, parental intention toward the influenza vaccine differed significantly between the development and validation groups.

**Table 1:** Characteristics of parents and their children in development and validation samples.

Variables	Group (1) development sample *N* = 430	Group (2) validation sample *N* = 2095	Test statistic *p*-value
	*n* (%)		
Mean age of mother	34.49 ± 7.8	36.53 ± 9.0	T = 6.38
			<0.001***
**Mother’s highest education level**			
Less than high school	49 (11.4)	417 (19.9)	𝜒2 = 21.76
High school	119 (27.7)	453 (21.6)	<0.001***
University or higher	262 (60.9)	1,225(58.5)	
**Mother’s employment status**			
Employed	179 (41.6)	1,196(57.1)	𝜒2 = 34.38
Unemployed	251(58.4)	899 (42.9)	<0.001***
**Place of residence**			
Urban	304 (70.7)	1,677 (80.0)	𝜒2 = 33.19
Rural	124 (28.8)	369 (17.6)	<0.001***
Mountains and desert	2 (0.5)	49(2.3)	
**Parents received influenza vaccination**			
Yes	147(34.2)	606(28.9)	𝜒2 = 4.72
No	283(65.8)	1,489(71.1)	0.018*
Average number of children	2.3 ± 1.2	3.17 ± 1.9	t = 10.116
			<0.001***
**Child’s age**			
Infant	90(20.9)	432(20.6)	𝜒2 = 7.63
Preschool	158(36.7)	641(30.6)	0.054
School children	88(20.5)	515(24.6)	
Adolescents	94(21.9)	507(24.2)	
**Child’s gender**			
Male	220(51.2)	1,131(54.0)	𝜒2 = 1.14
Female	210(48.8)	964(46.0)	0.155
Child ‘s birth order			
First	151(35.1)	524 (25.0)	𝜒2 = 85.46
Second-third	218(50.7)	791(37.8)	<0.001***
Forth or more	61(14.2)	780 (37.2)	
**Child had chronic disease**			
Yes	36(8.4)	234(11.2)	𝜒2 = 2.93
No	394(91.6)	1861(88.8)	0.049*
**Child got sick from influenza last year**			
Yes	247(57.4)	1,240(59.2)	𝜒2 = 33.89
No	86(20.0)	591(28.2)	<0.001***
Do not remember	97(22.6)	264(12.6)	
**Child got influenza vaccination last year**			
Yes	44 (10.2)	457(21.8)	𝜒2 = 30.08
No	386 (89.8)	1,638(78.2)	<0.001***
**Child got routine vaccination**			
Completely vaccinated	350(81.4)	1732(82.7)	𝜒2 = 6.16
Partially vaccinated	73(17.0)	291(13.9)	0.046*
Not vaccinated at all	7(1.6)	72 (3.4)	
**Child got COVID-19 vaccination**			
Yes	60(14.0)	220 (10.5)	𝜒2 = 4.31
No	370(86.0)	1875(89.5)	0.43
**Intention to vaccinate children**			
Yes	315(73.25)	1,361(65.0)	𝜒2 = 10.99
No	115(26.75)	734(35.0)	<0.001***

Age of children were categorized into infants: from 6 to 12 months, preschool age: 2–4 years, school age: 5–9 years, and adolescents: 10–18 years. The chi-square test was used to test differences between proportions, while the *t*-test is used to compare the means of the two groups.

* *p* < 0.05.

*** *p* < 0.001.

### 3.2. The model development

The null deviance of the prediction model that included only the intercept to quantify parents’ intention to vaccinate their children was 499.40. When fitting the parents’ intention model with all independent variables, including the PACV variable, the residual deviance was 360.27, and it was 432.62 if we excluded the PACV score. The Akaike Information criterion (AIC) was 410.27 if the PACV score was included and 480.62 if it was excluded. Including the fit of PACV improved the model and was highly significant (Table 2).

**Table 2:** The predictive PACV model.

Predictors	Coefficients (βs)	Odds Ratios (OR)	[95%CI]	*p*-value
(intercept)	0.152*	1.16	[1.06–1.90]	0.032
PACV score	−0.083***	0.92	[0.89–0.94]	<0.001
Mother’s age	0.030	1.03	[0.97–1.09]	0.315
working mother	0.374	1.45	[0.79–2.67]	0.226
Mother’s education: high school Mother’s education: university or higher	1.197 0.118	3.31 1.12	[0.26–4.49] [0.09–1.53]	0.337 0.924
Place of residence: rural	0.007	1.00	[0.54–1.89]	0.983
Parents received influenza vaccine the past year	0.308	1.36	[0.69–2.74]	0.377
Total number of children	0.074	1.08	[0.72–1.58]	0.703
Child age				
Preschool Schoolchildren Adolescents	0.004 −0.044 −1.796**	1.00 0.96 0.17	[0.44–2.28] [0.35–2.61] [0.05–0.54]	0.993 0.931 0.003
Child gender (Female)	0.271	1.31	[0.76–2.26]	0.328
Child’s birth order	−0.162	0.85	[0.61–1.24]	0.348
Child have a chronic disease	−0.264	0.77	[0.28–2.16]	0.610
Child get sick from influenza last yes	−0.431	0.85	[0.46–1.55]	0.600
Routine vaccination				
Child partially vaccinated Child not vaccinated at all	−0.273 −0.552	0.76 0.58	[0.32–1.78] [0.09–4.17]	0.527 0.561
Child get the COVID-19 vaccination	2.581***	1.32	[3.66–7.05]	<0.001
Child get influenza vaccination last year	0.436	1.55	[0.59–4.45]	0.390

Reference Categories. Mother education: less than high school. Place of residence: urban. Child age: infant. Routine vaccination: Completely vaccinated. Other variables are binary takes zero and one.Regression equation: ln (odds that parents intend to vaccinate their children) = 0.152-(0.083*PACV score) + (0.030*mother age) + (0.374*working mother) + (1.197*mother with high school) + (0.118 mother with university degree) + (0.007*rural parents) + (0.308*parents received vaccine) + (0.074*total number of children) + (0.004*preschool children) + (0.044*school children) − (1.796*adolescents) + (0.271*female child) − (0.162*birth order) − (0.264*child with a chronic disease) − (0.431*Child get sick from influenza) + (−0.273*child partially vaccinated) − (0.552*child not vaccinated at all) + (2.581*child get the COVI vaccination) + (0.436*child get inf vaccination).

* *p* < 0.05.

** *p* < 0.01.

*** *p* < 0.001.

### 3.3. Internal validation of the model

The proportion of parents’ intention that have been correctly classified was 79.3% [95% CI:75.5–83.1], while the classification error was 20.7% [95% CI:16.9–24.5]. The two cross-validation approaches, LOOCV and k-fold CV, yielded similar results, indicating that the PACV model had high predictive power, where the prediction error rate did not exceed 16.82%.

**Figure 1:**
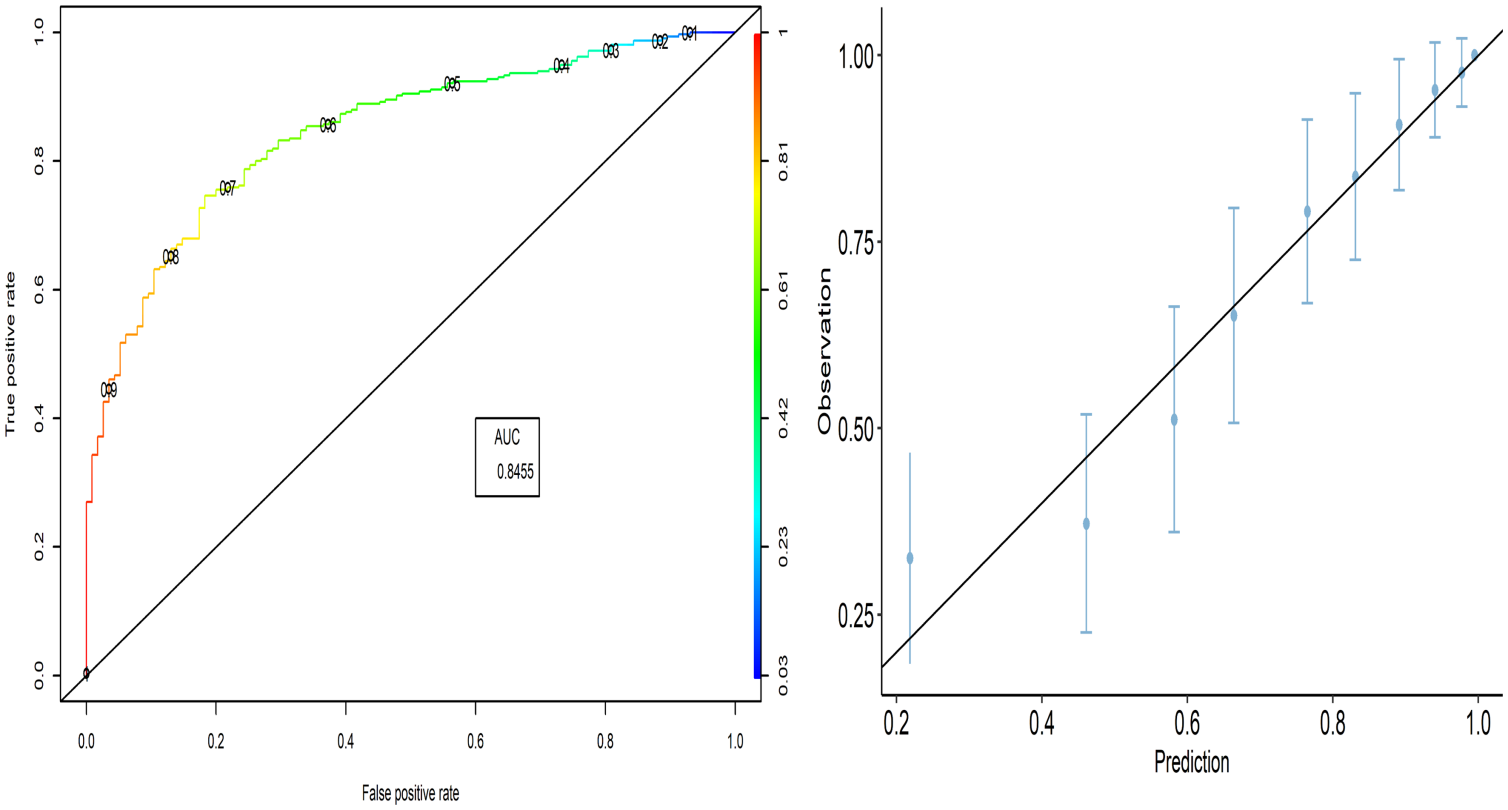
ROC curve and calibration plot for the PACV predictive model.

As shown in Figure 1, the AUC value was 0.846, indicating better performance of the predicted model. The calibration plot also demonstrates the accuracy of the calibration, where most points close to the diagonal line show better calibration. The TPR (sensitivity) was 92.06%, and the TNR (specificity) was 44.35%, respectively.

### 3.4. External validation

#### 3.4.1. Model discrimination

The external validity of the PACV model was evaluated using the model developed to predict the validation group. The PACV-developed model correctly predicted 69.4% [95% CI:67.8–70.8] of parents’ intention in the validation group, while its prediction error was 30.6% [95% CI:29.2–32.2]. Based on the confusion matrix, the PACV model incorrectly predicted 24.3% [95% CI:22.9–25.7] of parents in the intending group (Type I error) and 6.3% [95% CI:5.5–7.1] in the non-intending group, including parents who lack the intention of vaccination (Type II error). The TPR (sensitivity) was 89.64% [95% CI:88.6–90.6], and the TNR (specificity) was 37.9% [95% CI:36.3–39.5]. The ROC curve indicates that the TPR increases faster than the FPR, and the AUC value was 0.769, indicating the better performance of the predicted model.

#### 3.4.2. Model calibration

**Figure 2:**
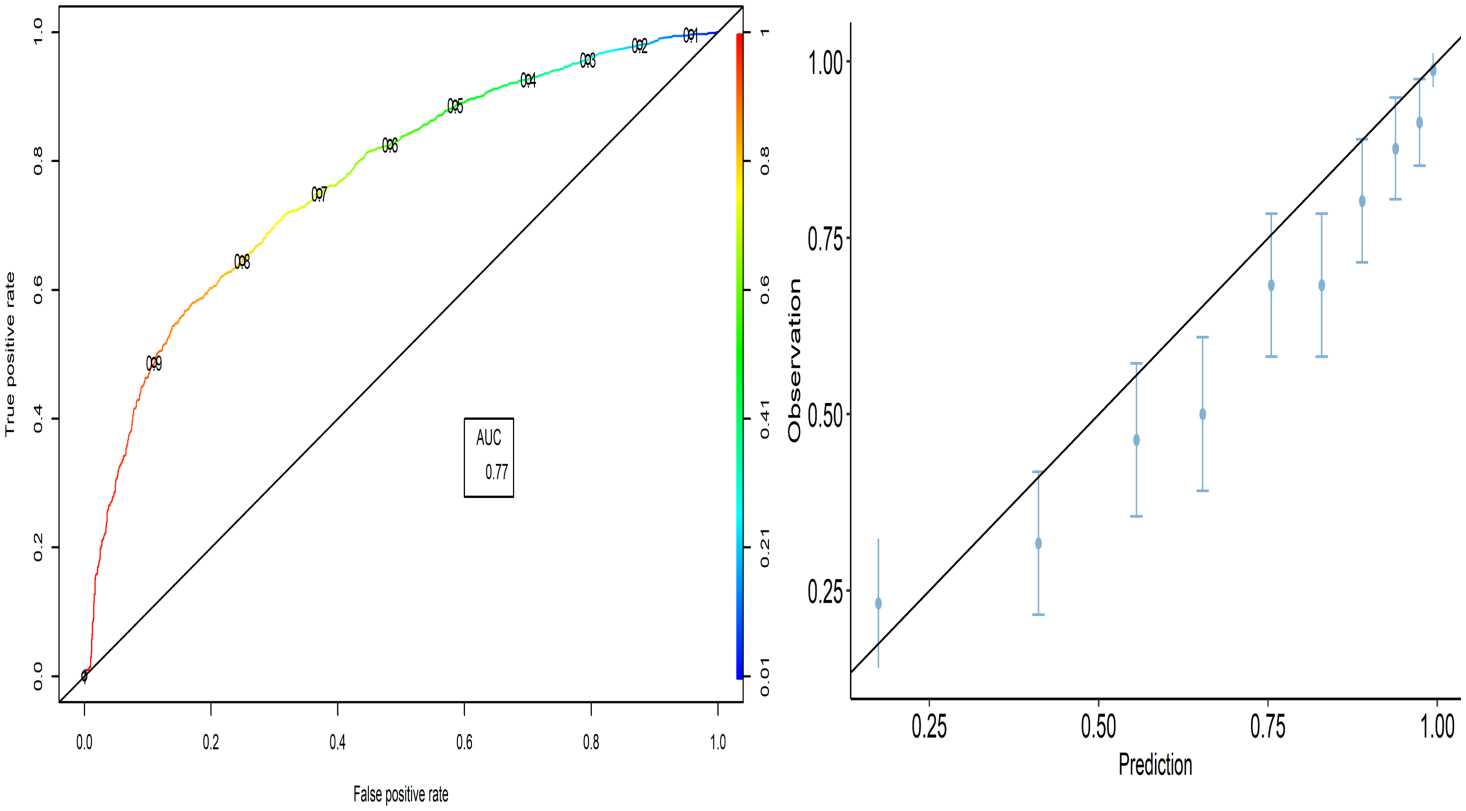
ROC curve and calibration plot for the validation group.

The calibration plot shows the validity of the predictive model, where most points are close to the diagonal line (45° line; Figure 2). Therefore, we can explore whether the PACV model performs better in all selected countries.

### 3.5. External validation across different countries

**Figure 3:**
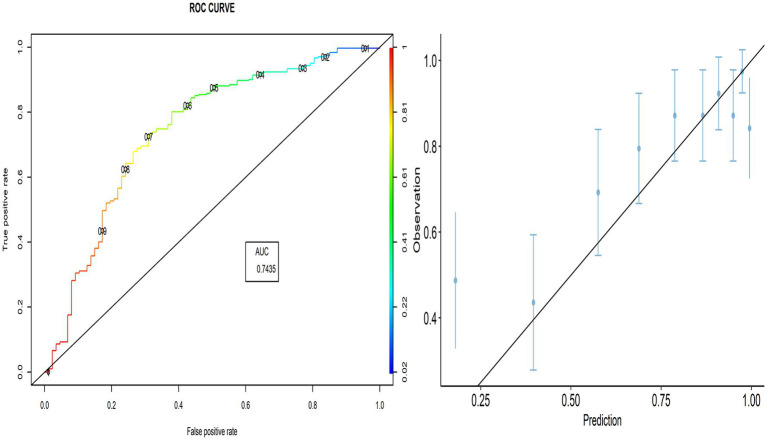
Roc curve and calibration plot for the PACV predictive model in Iraq.

**Figure 4:**
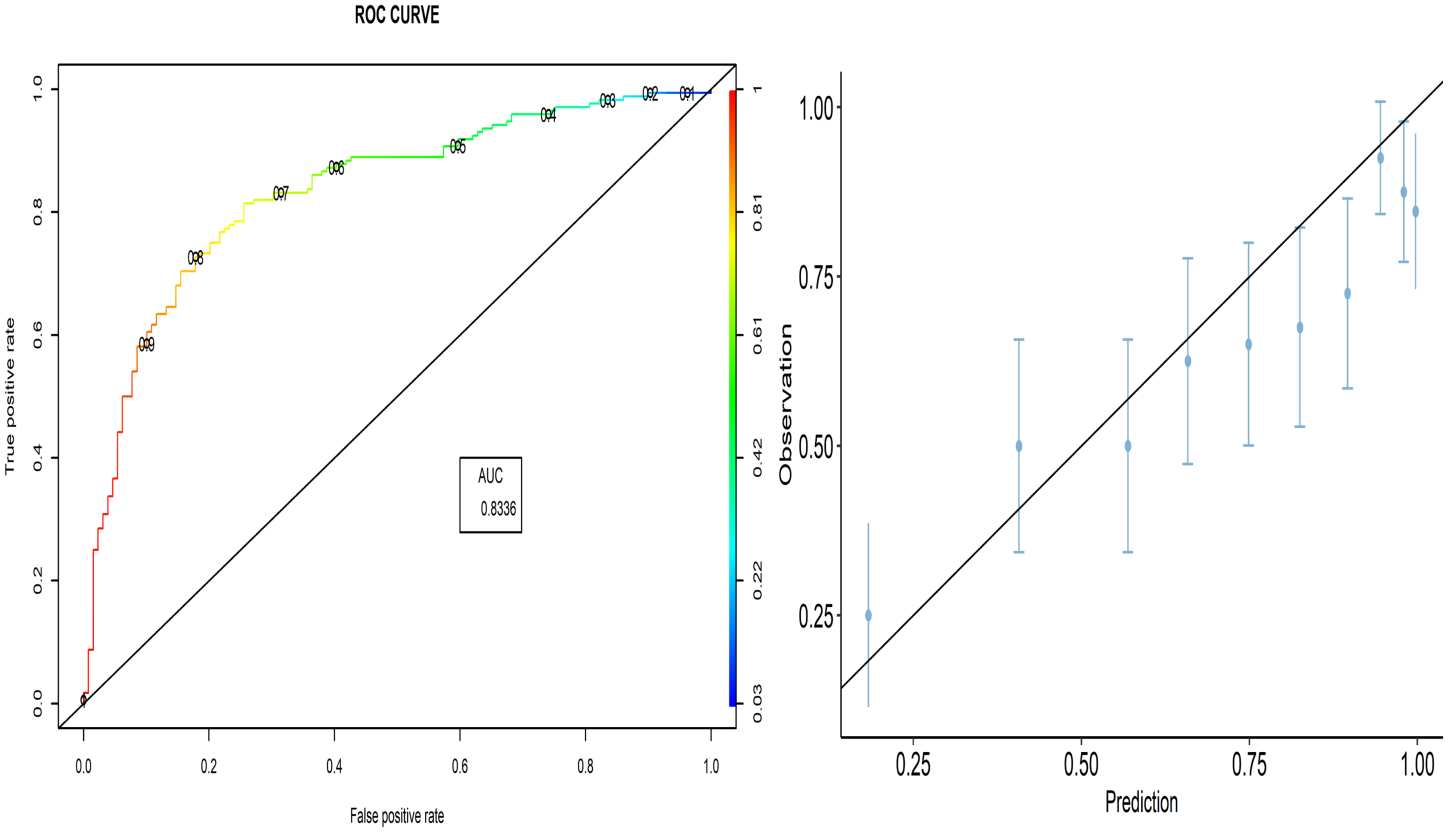
Roc curve and calibration plot for the PACV predictive model in Lebanon.

**Figure 5:**
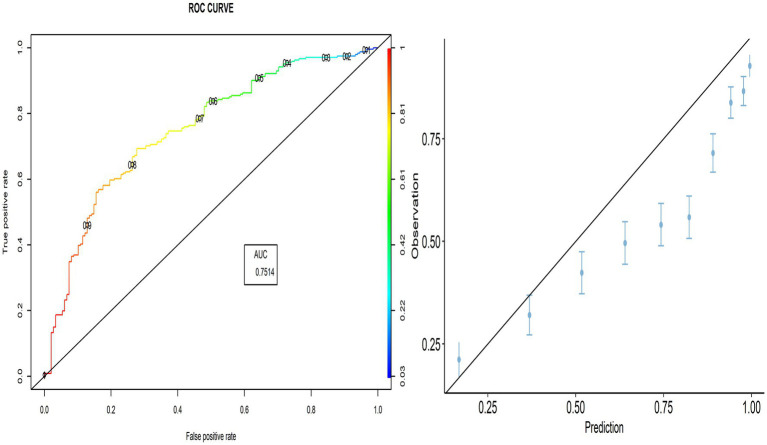
Roc curve and calibration plot for the PACV predictive model in Palestine.

**Figure 6:**
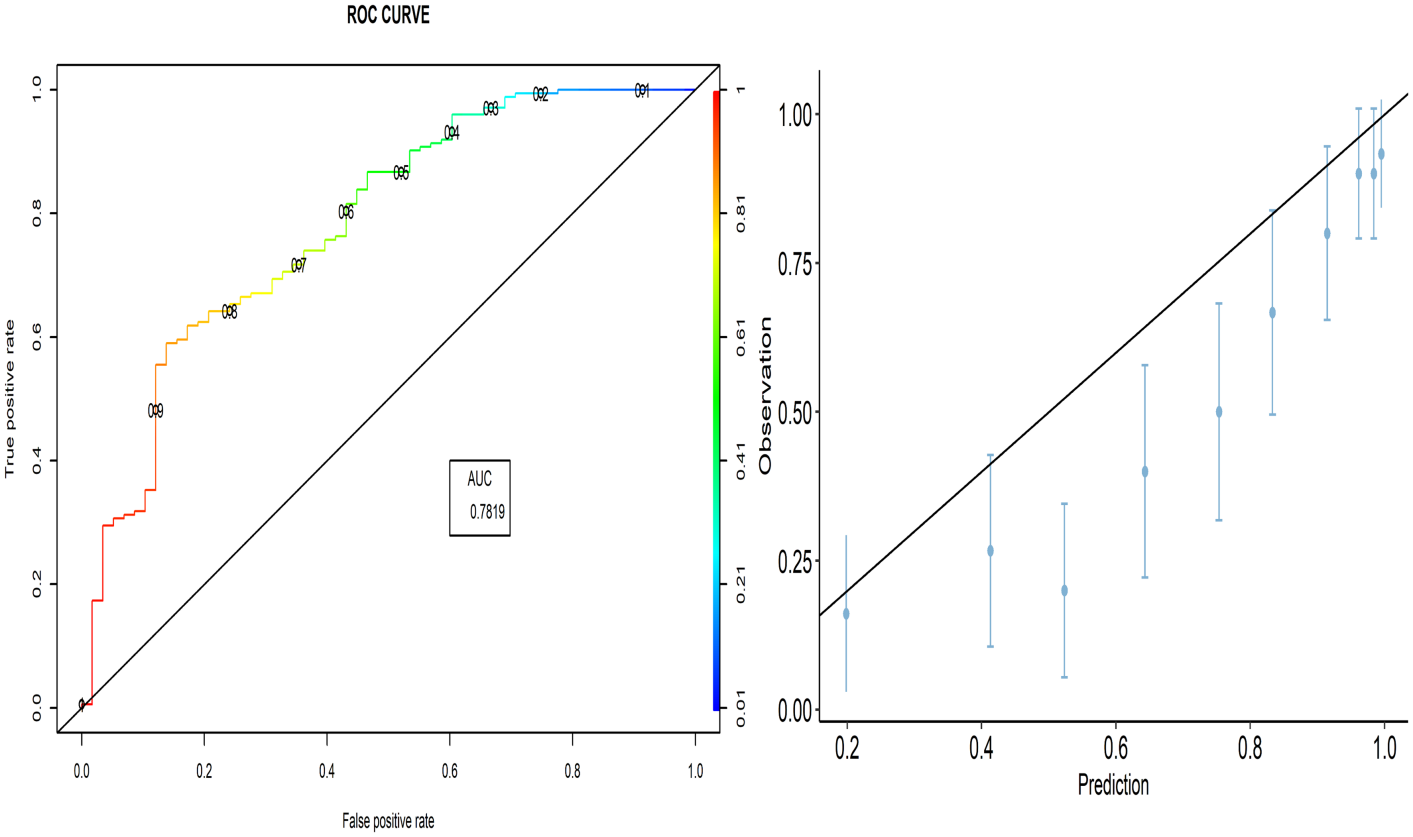
Roc curve and calibration plot for the PACV predictive model in Sudan.

**Figure 7:**
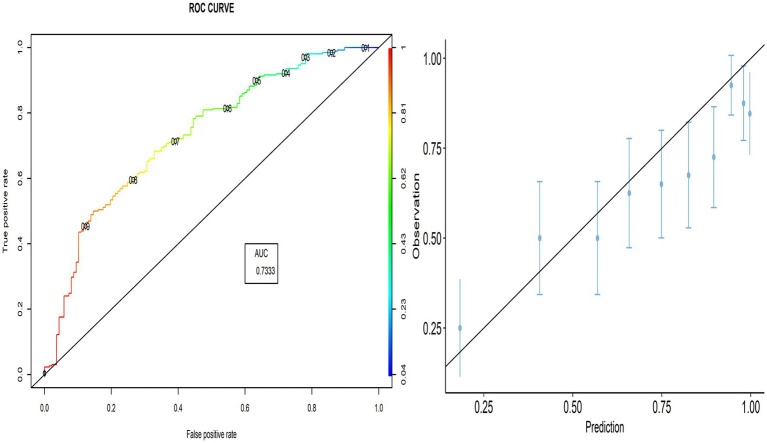
Roc curve and calibration plot for the PACV predictive model in Libya.

**Figure 8:**
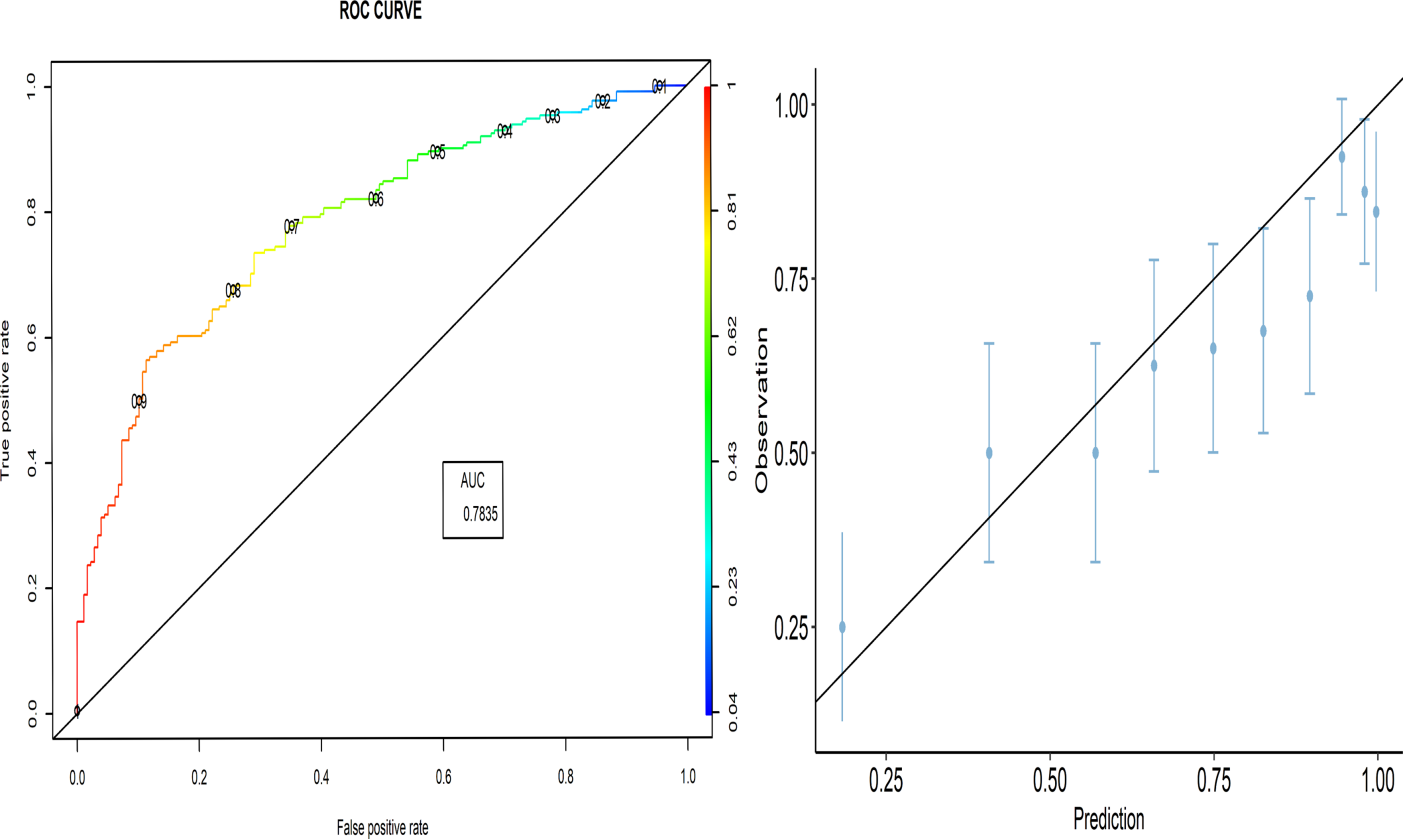
Roc curve and calibration plot for the PACV predictive model in Syria.

The proportion of correctly predicted observations differed across countries, as indicated in Table 3. The highest accuracy rate for the PACV predictive model was 78.92% in Iraq, and the lowest rate was 69.10% in Lebanon. Type I error was the highest in Palestine and the lowest in Iraq, whereas Type II error was the highest in Sudan and the lowest in Palestine. Figures 3–8 display the ROC curves and calibration plots for the PACV-predicted models. The model performs better as the AUC in all selected countries is large; the AUC has a greater value and is far from the diagonal line. Calibration plots indicated that the PACV prediction model performed better and had better predictive accuracy in the validation samples of the countries, where most points were close to the diagonal line.

**Table 3:** Performance assessment measures of the predictive classification model.

Country	Prediction accuracy rate	Prediction error rate	Type I error	Type II error	True-positive rate (Sensitivity)	True-negative rate (Specificity)	AUC
Libya	71.4% [67.0–75.9]	28.6% [24.1–33.0]	63.5% [58.8–68.2]	10.3% [7.3–13.3]	89.7% [86.7–92.7]	36.5% [31.8–41.2]	0.73 [0.68–0.77]
Lebanon	69.1% [63.9–74.3]	30.9% [25.7–36.1]	59.7% [54.1–65.2]	9.3% [6.2–12.6]	90.7% [87.4–94.0]	40.3% [3.5–45.9]	0.83 [0.79–0.84]
Syrian Arab Republic	67.6% [62.9–72.3]	32.4% [27.7–37.1]	58.9% [53.9–63.8]	10.5% [7.4–13.5]	89.6% [86.5–92.6]	41.1% [36.2–46.1]	0.78 [0.74–0.82]
Iraq	78.9% [74.9–83.0]	21.1% [17.0–25.1]	50.6% [45.6–55.5]	12.6% [9.3–15.9]	87.4% [84.12–90.7]	49.4% [44.5–84.1]	0.74 [0.70–0.79]
Palestine	69.9% [65.4–74.5]	30.1% [25.5–34.6]	64.2% [59.42–68.95]	9.1% [6.3–9.4]	90.9% [88.0–93.7]	35.1% [31.0–40.6]	0.75 [70.84–79.43]
Sudan	77.1% [71.6–82.5]	22.9% [17.5–28.4]	51.7% [45.3–58.2]	13.3% [8.9–17.7]	86.7% [82.3–91.1]	48.3% [41.83–54.7]	0.78 [0.72–0.83]

## 4. Discussion

With the increase in vaccine-preventable diseases, introduction of new vaccines, propagation of misinformation, and lack of coverage during the last decade, VH has been identified as a major public health challenge. This has resulted in a flood of scientific literature on VH in the realms of public health, biomedicine, and social science. Much of this research used different tools, such as the 5C scale and PACV, to assess VH. However, the overwhelming amount of data indicates that the quality of reporting in prediction model research is inadequate. This may provide incorrect information about the population’s attitude towards vaccination, and consequently affect the implementation of preventive measures to control infectious diseases. The implementation of these inappropriate measures may have deleterious effects on global health.

Furthermore, with the emergence of many infectious diseases such as coronavirus disease 2019 (COVID-19) and monkeypox, in addition to the ongoing risk of infection from circulating diseases such as seasonal influenza, there is a crucial need to develop and evaluate the validity of tools that assess VH among parents towards childhood vaccination. Although PACV has been used worldwide to assess parental VH, its external validity has not been examined. Therefore, the current study is the first to assess the external validity of the PACV scale across countries in two languages (Arabic and English) among 2095 participants from the general population.

Because of the unavailability of the equation of an internally validated model, we first developed the model, which was then externally validated. Internal validation of the model showed that PACV could effectively assess VH among parents. The model was developed in Egypt using a sample of 430 parents and it showed that PACV was a significant predictor of parents’ intention to vaccinate their children against influenza. In addition, the internal validity of the model showed good discrimination (AUC = 0.845, sensitivity = 92.06%, and specificity = 44.35%) and high predictive power (the prediction error rate did not exceed 16.82).

Unbiased validation of previous research findings is a fundamental scientific principle (28). After creating a prediction model, it is strongly recommended to test the model’s performance using data from individuals other than those used for model development. External validation involves using the previous model (the published regression formula) to make predictions for each individual in the new dataset and comparing them with the observed outcomes (16). Interestingly, many tools have been proven invalid when externally validated after being applied to thousands of patients. For instance, the Chronic Obstructive Pulmonary Disease (COPD) diagnostic questionnaire was created to improve the efficiency and precision of COPD diagnosis in primary care by distinguishing between individuals with and without airflow limitation. However, the COPD diagnostic questionnaire’s ability to differentiate between people with and without COPD was inadequate when externally validated, with an AUC of 0.65, sensitivity of 89.2%, and specificity of 24.24% (20). Similarly, Yu et al. (29) found that the vast majority of studies (70 of 86, 81%) that assessed the external validation of deep learning for radiologic diagnosis reported at least a decrease in external performance compared to internal performance. Almost half (42 of 86, 49%) reported a modest decrease (0.05 on the unit scale), and almost a quarter (21 of 86, 24%) reported a significant decrease (0.10 on the unit scale) in external performance compared with internal performance of the tool (29).

The external validation of a model depends primarily on its discrimination and calibration, which are essential for determining whether patients who experience an outcome have a higher expected risk than those who do not. For discrimination purposes, it makes little difference whether the absolute predicted risk is 8% or 80%, as long as the patient with the desired outcome has a higher risk (21).

In this study, external validity findings revealed that the discriminative ability of PACV was good. Moreover, the current study showed that the AUC of the six countries included in the external validation ranged from 70.3% in Libya to 83.3% in Lebanon. This result suggests that the model’s discrimination across countries with different economic incomes was acceptable.

A diagnostic test that can distinguish patients with and without a specific condition should have high sensitivity and specificity. The current study found that the sensitivity ranged from 86.7 to 90.9%, whereas the specificity ranged from 35.8 to 48.3%. This suggests that the developed model correctly identified nearly nine out of 10 parents intending to vaccinate their children against influenza (true-positive). At the same time, the questionnaire correctly classified nearly half of the participants as having no intention of getting their children vaccinated (true-negative).

TNR, which is the proportion of parents in the non-intending group who were correctly predicted by the developed model. The calibration of a model is defined as the degree of agreement between the predicted probabilities and observed outcomes (30). In the current study, the overall calibration and cross-country calibration of the model were good. This implies that the absolute expected outcome matches the observed risks. This finding is evident in the calibration plot of the entire dataset and at a country-based level.

Despite significant differences between countries in terms of mothers’ level of education, maternal age, place of residence, parents’ influenza vaccination, childbirth order, child status of having a chronic disease, received the influenza vaccine in the last year, the child received routine vaccination, and intention to receive influenza vaccination, PACV demonstrated good performance in diagnosing parental hesitancy about seasonal influenza vaccination. Accordingly, PACV can be used to identify VH among parents in different situations across different countries. Additionally, the current study emphasizes the importance of external validation of a newly designed diagnostic instrument before its inclusion in recommendations and clinical practice.

### 4.1 Strength and limitations

Although PACV has been widely used to assess parents’ attitudes toward different vaccines, its external validity has not been established until now. Therefore, our study is the first to evaluate the external validity of PACV among a large sample of the general population from six countries using both English and Arabic versions of the questionnaire.

However, this study had several limitations that should be considered in the future. First, the regression equation of the originally developed model was not available for use as a reference standard to compare our results. Therefore, we developed the model and tested its internal validity using the Egyptian dataset. Then, we used the developed equation to test the external validity of PACV on another larger dataset of six countries in the EMR. Second, the data were collected through an online survey. However, in most countries, web surveys have become the primary method of collecting data, surpassing face-to-face and computer-assisted telephone interviewing (CATI). This shift has been further accelerated by the COVID-19 pandemic, which has made traditional modes of data collection challenging. In fact, web surveys still encounter significant obstacles in obtaining probability-based samples that represent the general population, as a certain subset of the population with internet access and smartphones is targeted. However, according to 2022 statistics, a large population of the included countries had access to the Internet and were using social media platforms. For example, 94.5% of Egyptians had smartphones, nearly three-fourths of Egyptians had access to the Internet, and about 50% of them used social media platforms (31). Similar statistics have been reported in Iraq, where the number of social media users has increased by over 90%, as well as in Libya and Saudi Arabia (32). Third, the adopted web-based surveys rely on nonprobability survey designs, which are not considered the gold standard in survey sampling, unlike probability-based design. Nevertheless, nonprobability web surveys can still prove valuable in certain circumstances. For instance, nonprobability samples can help offset known biases in probability-based web survey samples by deliberately targeting underrepresented respondent profiles. In this study, we included a certain group of the population based on their representation (those living in mountains and deserts) to ensure the appropriate representation of all population categories. Additionally, to overcome the selection bias, we tried to include responses in proportion to each sector’s presentation despite using a non-probability sampling method. This is evident in Table 1, where there is nearly equal representation of different children age groups and parents’ working status in both the development and validation groups. Furthermore, we considered the population’s presentation based on their residence; most of respondents were living in urban areas, followed by rural areas, and then deserts and mountains. Finally, the cross-sectional survey itself has many inherent limitations, including the difficulty of determining whether the exposure or the outcome arrived first. Respondents’ recall and social acceptability biases are all examples of biases that might occur in the current study. Nonetheless, a cross-sectional design was the best design for addressing the study hypothesis.

## 5. Conclusion

Based on the findings of this study, the PACV model is a useful tool for assessing parental attitudes towards vaccination, regardless of the language used. The model has good discrimination and calibration, making it an effective tool for evaluating VH among parents in different countries. Policymakers and researchers can use the PACV model to assess and understand parental attitudes towards vaccination. Utilization of this tool can be extended to include pediatricians and other healthcare professionals. Identifying the determinants of parental attitudes is crucial, as it can help to increase vaccination acceptance and coverage by reducing VH. Therefore, we recommend the use of PACV as a valid tool to assess parents’ attitudes toward vaccination and to promote vaccination uptake among children.

## Data availability statement

The raw data supporting the conclusions of this article will be made available by the authors, without undue reservation.

## Ethics statement

The studies involving human participants were reviewed and approved by the Ethics Review Committee of Faculty of Medicine of Alexandria University (IRB no. 0305688). The patients/participants provided their written informed consent to participate in this study.

## Author contributions

RG and NF: conceptualization and methodology. RG, AE, SA-R, SA, and NY: data analysis. RG, AE, SA-R, and SA: data curation. RG, ST, and SE: writing—original draft preparation. RG, ST, SE, NY, and NF: writing—review and editing. RG, SA, TA, and BK: visualization. All authors contributed to the article and approved the submitted version.

## Conflict of interest

The authors declare that the research was conducted in the absence of any commercial or financial relationships that could be construed as potential conflicts of interest.

## Publisher’s note

All claims expressed in this article are solely those of the authors and do not necessarily represent those of their affiliated organizations, or those of the publisher, the editors and the reviewers. Any product that may be evaluated in this article, or claim that may be made by its manufacturer, is not guaranteed or endorsed by the publisher.

## Abbreviations

AIC: Akaike Information criteria
AUC: Area under the curve
COPD: Chronic obstructive pulmonary disease
COVID-19: Coronavirus disease 2019
EMR: East Mediterranean Region
LOOCV: Leave-one-out cross-validation
PACV: Parental attitudes about childhood vaccines
PI: Prognostic index
ROC: Receiver operating characteristic
TNR: True-negative rate
TPR: True-positive rate
FPR: False-positive rate
VH: Vaccine hesitancy
