# Supplementary material for: External validation of the parental attitude about childhood vaccination scale
Source: Front Public Health. 2023 May 15;11:1146792. doi: 10.3389/fpubh.2023.1146792 (PMC10228384; doi:10.3389/fpubh.2023.1146792)
Supplement: Supplementary file 1 [file Data_Sheet_1.docx]

**Data collection tool (Arabic and English versions)**

أنتم مدعوون للمشاركة في بحث يحمل عنوان "موقف وممارسة الآباء والأمهات اتجاه تلقى اطفالهم لقاح الانفلونزا الموسمي". يهدف هذا البحث الى تقييم موقف وممارسة الآباء والأمهات موقفهم اتجاه تلقى ابنائهم لقاح الانفلونزا الموسمي. يستهدف هذا البحث الآباء والأمهات الذين لديهم ابناء تتراوح اعمارهم بين ستة أشهر حتى 18 سنة. نتائج البحث الحالي قد تتيح الفرصة لوضع خطة توعية حول لقاح الانفلونزا الموسمي بهدف رفع مستوى تلقيح الاطفال وبالتالي خفض معدل الأمراض المعدية بين الأطفال. مشاركتكم في هذا الاستبيان طوعيّ ولكم كل الحرية في الانسحاب من هذه الدراسة في أي وقت ترغبون بذلك. ولقد تمت الموافقة على هذه الدراسة من قبل لجنة الأخلاقيات بكلية الطب ورقمها 00012098)). وستكون جميع البيانات مجهولة المصدر دون اية اشارة لهويتك الشخصية.

You are invited to participate in research entitled" Parental attitude and practice towards seasonal influenza vaccine among their children". Our aim is to assess parents' attitude and practice towards vaccinating their children against seasonal influenza vaccine. This research targets parents who have children aged between six months and up to 18 years. Results of our research could provide an opportunity to develop awareness campaigns about the seasonal influenza vaccine in order to increase the level of vaccination among children and subsequently reduce the rate of infectious diseases among children. Your participation is voluntary, and you are free to withdraw at any time. This study was approved by the Ethics Committee of the Faculty of Medicine with IRB number (00012098). Confidentiality will be maintained.

- Please select the language: :برجاء اختيار اللغة المناسبة لك

1. اللغة العربية
2. English
3. **بيانات ديموغرافية**
4. **من يقوم بمليء الاستبيان ؟**
   1. الام
   2. الاب
   3. آخرون:
5. **بلد الإقامة**
   1. مصر
   2. ليبيا
   3. لبنان
   4. السودان
   5. سوريا
   6. العراق
   7. فلسطين
6. **مكان السكن:**
   1. مدينة/حضر
   2. ريف
   3. جبال / صحراء
   4. اخري:..
7. **عدد الاطفال في الاسرة: -----**
8. **سن الام (السنوات): -----**
9. **اعلى شهادة او درجة تعليمية حصلت عليها الام**:
   1. لا يوجد
   2. شهادة التعليم الابتدائي
   3. اقل من الشهادة الثانوية
   4. شهادة التعليم الثانوي /البكالوريا
   5. شهادة مهنية/ تقنية
   6. شهادة جامعية
   7. دراسات عليا
10. **وظيفة الام**:
    1. تعمل
    2. لا تعمل
11. **هل تلقى احد الوالدين او كلاهما لقاح الانفلونزا الموسمي خلال العام الماضي؟**
    1. نعم
    2. لا
12. **معلومات عن الطفل (الفئة العمرية من 6 اشهر حتى 18 سنة)**

اذا كان لديك اكثر من طفل واحد في الفئة العمرية (6 اشهر حتى 18 سنة) برجاء استكمال الاستبيان بناء على الطفل الاصغر / الاخير

1. **سن الطفل**
   1. 6 اشهر
   2. 7 اشهر
   3. 8 اشهر
   4. 9 اشهر
   5. 10شهر
   6. 11 اشهر
   7. 1 سنة
   8. 2 سنوات
   9. 3 سنوات
   10. 4 سنوات
   11. 5 سنوات
   12. 6 سنوات
   13. 7 سنوات
   14. 8 سنوات
   15. 9 سنوات
   16. 10 سنوات
   17. 11 سنة
   18. 12 سنة
   19. 13 سنة
   20. 14 سنة
   21. 15 سنة
   22. 16 سنة
   23. 17 سنة
   24. 18 سنة
2. **جنس الطفل**
   1. ذكر
   2. أنثى
3. **ترتيب الطفل بين اخواته: -----**
4. **هل يعاني طفلك من أي امراض مزمنة (أي يتطلب منه تناول ادوية بشكل مستمر مثل امراض حساسية صدر - سكرى- القلب. الخ*.)***
   1. نعم
   2. لا
5. **هل اصيب طفلك بالأنفلونزا العام الماضي؟**
   1. نعم
   2. لا
   3. لا اتذكر
6. **هل تلقى طفلك كل اللقاحات الاجبارية/ الالزامية؟**
   1. نعم
   2. حصل على بعض منها
   3. لا لم يتلقى أي منها
7. **هل تلقى طفلك لقاح كوفيد-19 (كورونا)؟**
   1. نعم
   2. لا
8. **هل تلقى طفلك لقاح الانفلونزا العام الماضي؟**
   1. نعم
   2. لا
9. **هل لديك النية ليتلقى طفلك لقاح الانفلونزا العام القادم؟**
   1. نعم
   2. لا
10. **ممارسات وسلوك الآباء والأمهات تجاه لقاح الانفلونزا**
11. **هل سبق لك في أي وقت أن أجلت تطعيم طفلك لقاح الانفلونزا لأسباب غير المرض أو الحساسية؟**
    1. نعم
    2. لا
    3. لا اعلم
12. **هل سبق لك أن قررت الامتناع عن تطعيم طفلك لقاح الانفلونزا لأسباب غير المرض أو الحساسية؟**
    1. نعم
    2. لا
    3. لا اعلم
13. **إلى أي مدى أنت متأكد أن الالتزام بجرعات لقاح الانفلونزا الموصي بها سنوياَ مفيدة بالنسبة لطفلك**
    1. 1 2 3 4 5 6 7 8 9 10

غير متأكد على الاطلاق متأكد جدا

1. **اذا رزقت بطفل في الوقت الحالي هل ترغب بأن يتلقى لقاح الانفلونزا؟**
   1. نعم
   2. لا
   3. لا اعلم
2. **عموما، الى أي درجة تعتبر نفسك متردد بشأن لقاح الانفلونزا للأطفال ؟**
   1. غير متردد على الاطلاق
   2. غير متردد نوعا ما
   3. غير متأكد
   4. متردد نوعاَ ما
   5. متردد جداَ
3. **يتلقى الأطفال عدد أكبر من جرعات لقاح الانفلونزا اللازمة لهم**
   1. أوافق بشدة
   2. أوافق
   3. غير متأكد
   4. لا أوافق
   5. لا اوافق بشدة
4. **أعتقد أن لقاح الانفلونزا يقي من الأمراض الشديدة**
   1. أوافق بشدة
   2. أوافق
   3. غير متأكد
   4. لا أوافق
   5. لا اوافق بشدة
5. **من الأفضل لطفلي أن يكتسب مناعة عن طريق الاصابة بالمرض بدلا من أخذ لقاح الانفلونزا**
   1. أوافق بشدة
   2. أوافق
   3. غير متأكد
   4. لا أوافق
   5. لا اوافق بشدة
6. **من الأفضل أن يحصل الأطفال على عدد قليل من جرعات لقاح الانفلونزا**
   1. أوافق بشدة
   2. أوافق
   3. غير متأكد
   4. لا أوافق
   5. لا اوافق بشدة
7. **الى أي مدى تشعر بالقلق من أن يسبب لقاح الانفلونزا عرض جانبي خطير لطفلك ؟**
   1. غير قلق اطلاقا
   2. غير قلق نوعا ما
   3. غير متأكد
   4. قلق نوعا ما
   5. قلق جداَ
8. **الى أي مدى تشعر بالقلق أن يكون لقاح الانفلونزا غير آمن للاطفال ؟**
   1. غير قلق اطلاقا
   2. غير قلق نوعا ما
   3. غير متأكد
   4. قلق نوعا ما
   5. قلق جداَ
9. **إلى أي مدى تشعر بالقلق أن لقاح الانفلونزا لن يقي من المرض؟**
   1. قلق اطلاقا
   2. غير قلق نوعا ما
   3. غير متأكد
   4. قلق نوعا ما
   5. قلق جداَ
10. **اثق بالمعلومات التي اتلقاها عن لقاح الانفلونزا**
    1. أوافق بشدة
    2. أوافق
    3. غير متأكد
    4. لا أوافق
    5. لا اوافق بشدة
11. **أنا قادر على مناقشة مخاوفي الخاصة بلقاح الانفلونزا مع طبيب الأطفال**
    1. أوافق بشدة
    2. أوافق
    3. غير متأكد
    4. لا أوافق
    5. لا اوافق بشدة
12. **اجمالا، الى أي مدى تثق في طبيب الأطفال؟**
    1. 1 2 3 4 5 6 7 8 9 10

لا اثق اطلاقا أثق جدا

1. **Sociodemographic data**
2. **Who is filling the questionnaire?**
   1. Mother
   2. Father
   3. Other:
3. **Country**
   1. Egypt
   2. Iraq
   3. Lebanon
   4. Libya
   5. Palestine
   6. Sudan
   7. Syrian Arab Republic
4. **residence**
   1. Urban/ city
   2. Rural
   3. Desert region/ mountains
   4. Other:
5. **Total number of children: -----**
6. **Mother's age (years): -----**
7. **What is the highest degree or level of school the mother have completed? ***
   1. Primary school graduate
   2. Less than high school
   3. High school graduate
   4. Technical school graduate
   5. University/college
   6. Postgraduate
8. **Employment of the mother**
   - - 1. Employed
       2. Not employed
9. **Did one or both parents receive the influenza vaccine within the past year?**
   1. Yes
   2. No
10. **Child’s information (child aged from 6 months up to 18 years old is included)**

If you have more than one child in the target age group (6 months to 18 years), you have to complete the survey based on the youngest child.

1. **Age of the child**
   1. 6 months
   2. 7 months
   3. 8 months
   4. 9 months
   5. 10 months
   6. 11 months
   7. 1 year
   8. 2 years
   9. 3 years
   10. 4 years
   11. 5 years
   12. 6 years
   13. 7 years
   14. 8 years
   15. 9 years
   16. 10 years
   17. 11 years
   18. 12 years
   19. 13 years
   20. 14 years
   21. 15 years
   22. 16 years
   23. 17 years
   24. 18 years
2. **Gender of the child**
   1. Male
   2. Female
3. **Birth order of the child: -----**
4. **Does your child suffer from any chronic illness that requires him/her to take medication on a regular basis (Diabetes, asthma, etc) ?**
   1. Yes
   2. No
5. **Did your child get sick from influenza last year?**
   1. Yes
   2. No
   3. I don’t remember
6. **Did your child get the routine vaccination?**
   1. Completely vaccinated up to age
   2. Partially vaccinated
   3. Not vaccinated at all
7. **Did you vaccinate your child for COVID-19 ?**
   1. Yes
   2. No
8. **Did you vaccinate your child for influenza last year?**
   1. Yes
   2. No
9. **Do you have the intention to vaccinate your child for influenza?**
   1. Yes
   2. No
10. **Parental attitude and practice towards influenza vaccination among thier children**
11. **Have you ever delayed having your child get influenza vaccine for reasons other than illness or allergy?**
    1. Yes
    2. No
    3. I do not know
12. **Have you ever decided not to have your child get influenza vaccine for reasons other than illness or allergy?**
    1. Yes
    2. No
    3. I do not know
13. **How sure are you that following the recommended shots of influenza vaccine is a good idea for your child**

1 2 3 4 5 6 7 8 9 10

Not sure at all Completely sure

1. **If you had another infant today, would you want him/her to get all the recommended shots of influenza vaccine?**
   1. Yes
   2. No
   3. I do not know
2. **Overall, how hesitant about childhood shots of influenza vaccine would you consider yourself to be?**
   1. Not hesitant at all
   2. Not too hesitant
   3. Not sure
   4. Somewhat hesitant
   5. Very hesitant
3. **Children get more shots of influenza vaccine than are good for them.**
   1. Strongly agree
   2. Agree
   3. Not sure
   4. Disagree
   5. Strongly disagree
4. **I believe that influenza vaccine prevent many of severe illnesses**
   1. Strongly agree
   2. Agree
   3. Not sure
   4. Disagree
   5. Strongly disagree
5. **It is better for my child to develop immunity by getting sick than to get a shot of influenza vaccine Mark only one oval.**
   1. Strongly agree
   2. Agree
   3. Not sure
   4. Disagree
   5. Strongly disagree
6. **It is better for children to get fewer shots of influenza vaccine**
   1. Strongly agree
   2. Agree
   3. Not sure
   4. Disagree
   5. Strongly disagree
7. **How concerned are you that your child might have a serious side effect from * influenza vaccine?**
   1. Not concerned at all
   2. Not too concerned
   3. Not sure
   4. Somewhat concerned
   5. Very concerned
8. **How concerned are you that influenza vaccine might not be safe for children?**
   1. Not concerned at all
   2. Not too concerned
   3. Not sure
   4. Somewhat concerned
   5. Very concerned
9. **How concerned are you that influenza vaccine might not prevent the disease?**
   1. Not concerned at all
   2. Not too concerned
   3. Not sure
   4. Somewhat concerned
   5. Very concerned
10. **I trust the information I receive about influenza vaccine ***
    1. Strongly agree
    2. Agree
    3. Not sure
    4. Disagree
    5. Strongly disagree
11. **I am able to openly discuss my concerns about influenza vaccine with my child’s doctor**
    1. Strongly agree
    2. Agree
    3. Not sure
    4. Disagree
    5. Strongly disagree
12. **All things considered, how much do you trust your child’s doctor?**

1 2 3 4 5 6 7 8 9 10

Do not trust at all Completely trust
