# Supplementary material for: External validation of the parental attitude about childhood vaccination scale
Source: Front Public Health. 2023 May 15;11:1146792. doi: 10.3389/fpubh.2023.1146792 (PMC10228384; doi:10.3389/fpubh.2023.1146792)
Supplement: Supplementary file 3 [file Data_Sheet_3.doc]

**The Predictive PACV model**

We investigated the linear relationship between the PACV score, the mother's age, the number of children, birth order, and the logit of 'the parents' intention outcome. The smoothed scatter plots indicated that all continuous variables are quite linearly associated with 'parents' intention in the logit scale (Fig S.1). Cook's distance and standardized residuals are measured to check the influential values. Fig S.2 shows the most extreme observations based on Cook's distance. Still, there were no influential observations because their absolute standardized residuals were less than 3 as indicated in Fig S.3. Moreover, our model did not suffer from a multicollinearity problem as all independent variables have a value of variance inflation factor less than 5.


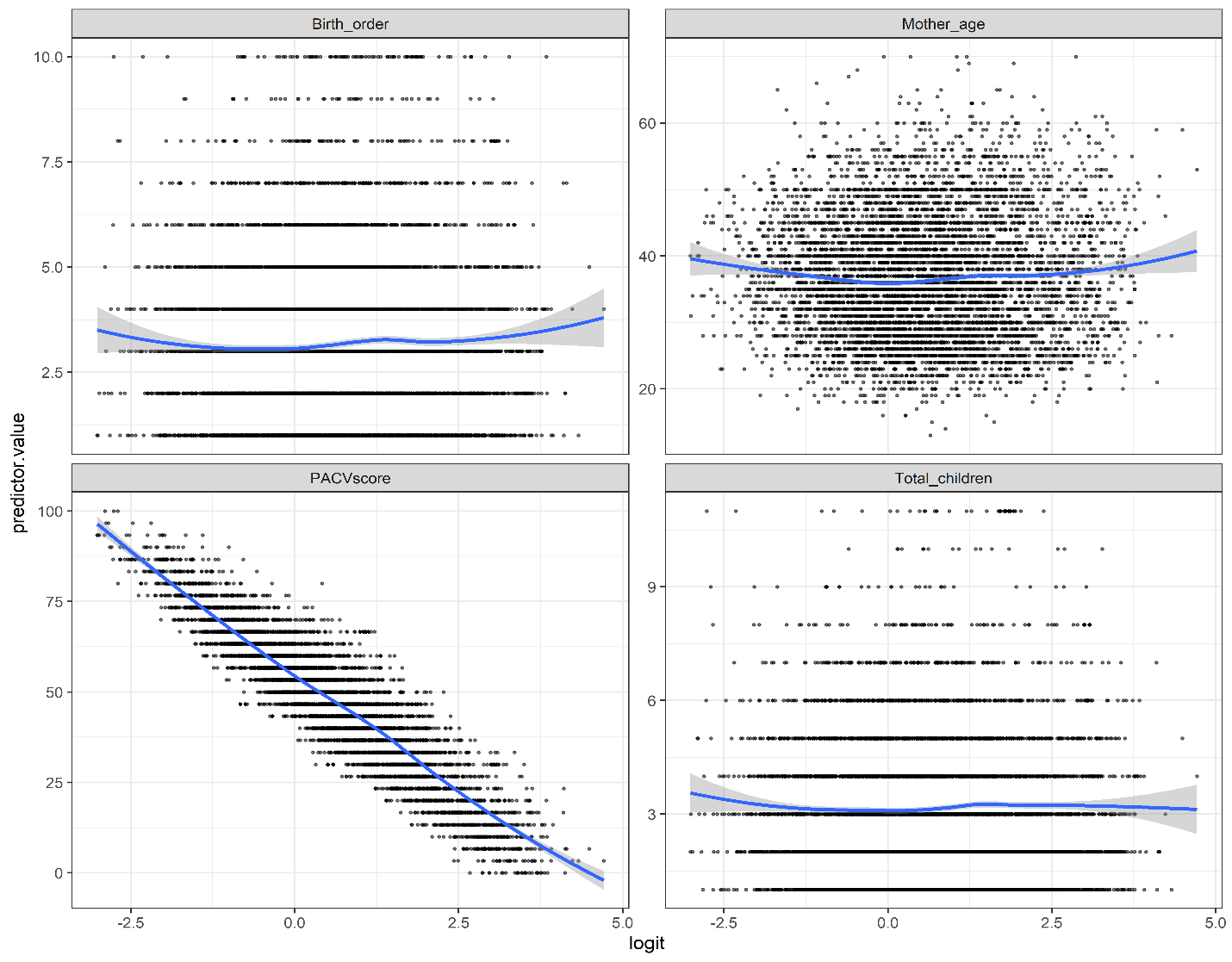


Fig S.1 Smoothed scatter plot for relations between continuous variables and logit 'parents' intention


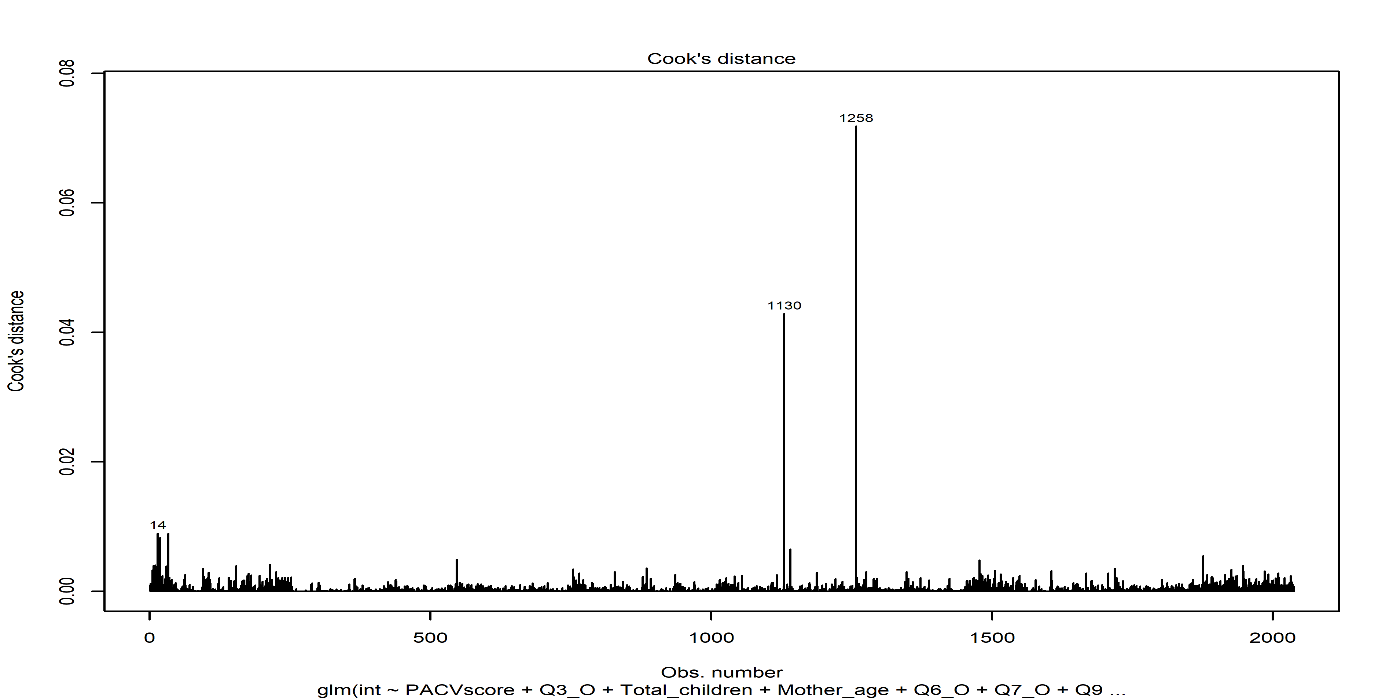


Fig S.2. Cook's distance values for extreme outliers


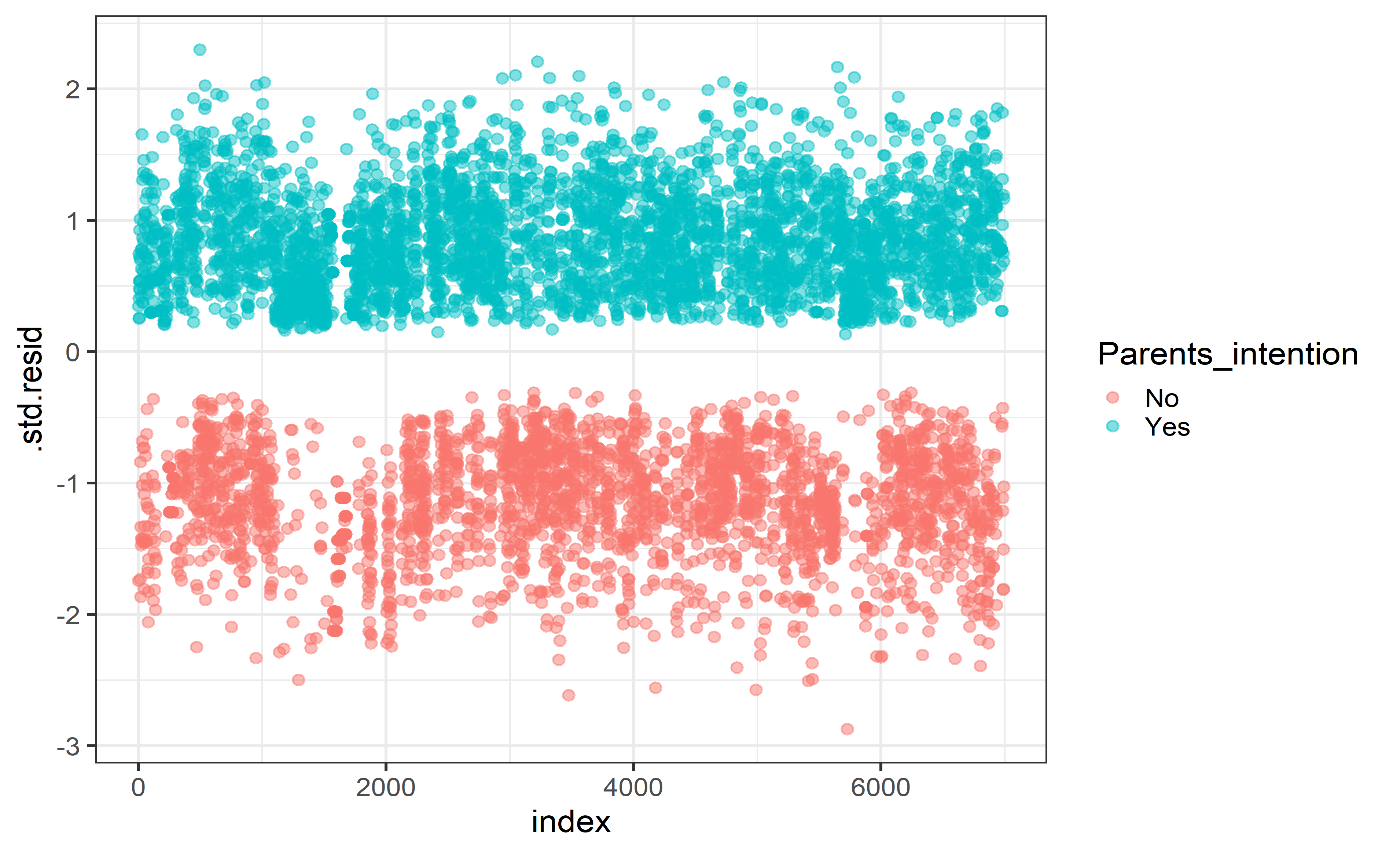


Fig S.3. Standardized residuals

**Cross Validation techniques**

**The LOOCV approach** divides the data into two samples: the development sample and the validation sample. But unlike the classic approaches, LOOCV does not divide the data into two comparable samples where the model is fit on the
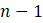
 development observations and the excluded observation is used for predicting the outcome. Repeating the procedure
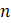
 times will produce
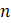
 prediction errors. **LOOCV** estimate is the average of these n prediction errors as follows: -


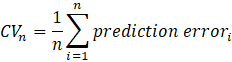


Prediction errors refer to misclassified observations. LOOCV is an unbiased technique and avoids overestimating prediction errors by adjusting the model
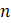
 times. Moreover, applying LOOCV multiple times will produce the same results because it does not randomly split the data.


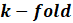
 **CV** approach randomly splits the data into
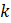
 equal size folds. The first fold is used as a validation group and the remaining (
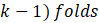
 used as the development group, and then the procedure is performed
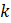
 times and will produce
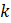
 prediction errors.
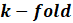
 **CV** estimate is the average of these
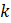
 values as follows: -


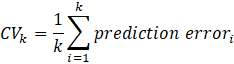


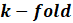
 **CV** approach is considered the general case for the LOOCV approach, where
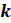
 equals
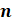
. Additionally,
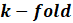
 **CV** is distinguished from LOOCV in reducing computations, but neither is superior to the other. We performed the most common number of the fold (
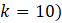
 because it results in prediction errors that are less subjected to high bias or high variance. Therefore, we randomly split the data into ten folds. We found that the results did not vary across the drawn samples.
